# Can pupillometry diagnose a lack of analgesia prior to nursing in critically ill patients?

**DOI:** 10.1186/2197-425X-3-S1-A326

**Published:** 2015-10-01

**Authors:** T Gaillard, S Gergaud, CM Grayot, C François, JF Hamel, S Lasocki

**Affiliations:** Univ. Hospital Angers, Anesthésie Réanimation, Angers, France; Univ. Hospital Angers, Centre de Recherche Clinique, Angers, France

## Introduction

Patient's nursing is carried out several time per day in the intensive care. It is often described as painful by patients. The measurement of pupillary dilatation reflex (PDR) during a tetanic stimulation by pupillometry predicts analgesia level in the operating room and during tracheal aspirates in critically ill patients [1].

## Objectives

The objective of this study is to evaluate whether this PDR predicts insufficient analgesia prior to a nursing care.

## Methods

Prospective, interventional study conduct in an academic surgical ICU. After local ethic committee agreement and consent (relatives and patients)

Critically ill patients, intubated and mechanically ventilated, sedated, were included in the absence of ocular pathology, pace-maker, neuromuscular blockade agent, TBI or spinal cord injury. Before each nursing, the PDR was measured using a pupilometer (AlgiScan, IDMed France) during tetanic stimulation at increasing intensity (5, 10, 20, 40 and 60 mA). The PPI score was also measured, it combines automated increasing tetanic stimulations with measurement of PDR, quoted in a scale ranging from 0 to 10. During each nursing care pain was measured using the Behavorial Pain Scale (BPS). PDR values for each stimulation threshold and PPI score were compared for painful (BPS> 5) and not painful (BPS≤5) nursing cares. The best diagnostic value was determined using ROC curves.

## Results

**Figure 1:**
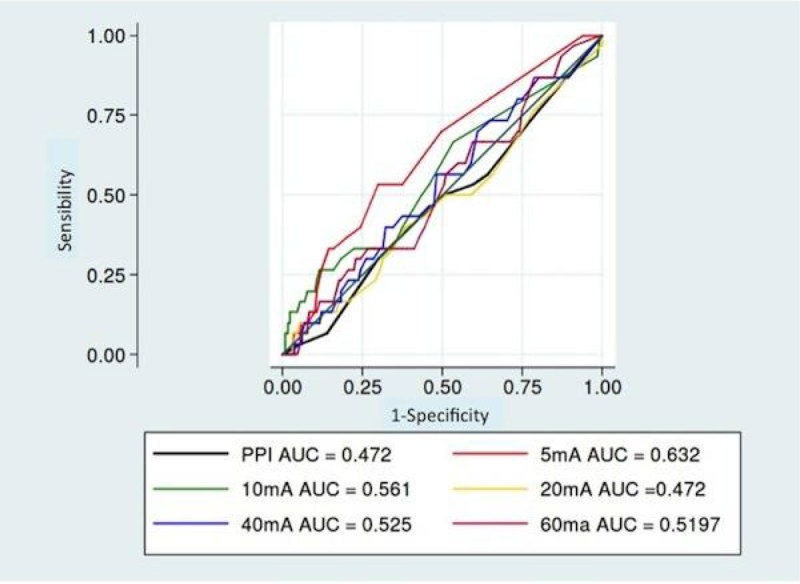
Figure 1

Among the 170 nursings achieved in 41 patients (age 65 ± 15 years, 33 (80%) men, SAPSII 56 ± 18, 82% emergent surgeries, 5% planned surgeries), 32 (18.8%) were painful. Blood pressure during the nursing care were higher during painful nursings (p = 0.02). But no other parameters were associated with pain during nursing (including the doses of sedation and analgesia). The areas under the curves of PDR at different stimulation levels did not exceed 0.6 (Figure 1), with no difference between the different intensity of stimulations. The PPI score was not better (4.1[3.2 to 5] vs 4.9[4.5 to 5.4] for painful or not, p = 0.10).

## Conclusions

Pupillometry can not predict insufficient analgesia prior to a nursing care in surgical ICU patients. The heterogeneity of diseases (peritonitis, mediastinitis, multiple trauma, medical cause...) could explain these results, the same procedure (ie a nursing care) may induce an highly variable degree of pain depending on the patient conditions.
